# Cranial autonomic symptoms in migraine are related to central sensitization: a prospective study of 164 migraine patients at a tertiary headache center

**DOI:** 10.1186/s12883-022-02610-8

**Published:** 2022-03-14

**Authors:** Daisuke Danno, Johanna Wolf, Kumiko Ishizaki, Shoji Kikui, Koichi Hirata, Takao Takeshima

**Affiliations:** 1 Headache Center and Department of Neurology, Tominaga Hospital, 1 - 4 - 48 Minatomachi, Naniwa ward, Osaka, Japan; 2 Division of Neurology, Department of Internal Medicine, Hyogo College of Medicine, Nishinomiya, Hyogo Japan; 3 Department of Neurology, Dokkyo medical university, Tochigi, Japan

**Keywords:** Migraine, Cranial autonomic symptom, Cranial autonomic parasympathetic symptom, Central sensitization, Allodynia, Aural fullness, Central sensitization inventory

## Abstract

**Background:**

Cranial autonomic symptoms (CASs) during migraine attacks are reported to be quite common regardless of ethnicity. In our previous study investigating 373 migraineurs, we found that 42.4% of them had CASs. The patients with CASs more frequently had cutaneous allodynia than did those without CASs, and we speculated that CASs were associated with central sensitization. The present study searched for substantial evidence on the relationship between CASs and central sensitization in migraine patients.

**Methods:**

This was a prospective cross-sectional study. We studied a new independent cohort of 164 migraineurs who presented to the Tominaga Hospital Headache Center from July 2018 until December 2019. The clinical features of CASs according to the criteria in ICHD-3 (beta) were investigated. We also evaluated central sensitization based on the 25 health-related symptoms utilizing the validated central sensitization inventory (CSI), and each symptom was rated from 0 to 4 resulting a total score of 0–100.

**Results:**

The mean age was 41.8 (range: 20 to 77) years old. One hundred and thirty-one patients (78.9%) were women. Eighty-six of the 164 (52.4%) patients had at least 1 cranial autonomic symptom. The CSI score of the patients with ≥3 CASs reflected a moderate severity and was significantly higher than in those without CASs (41.9 vs. 30.7, *p* = 0.0005). The score of the patients with ≥1 conspicuous CAS also reflected a moderate severity and was significantly higher than in those without CASs (40.7 vs. 33.2, *p* = 0.013). The patients in the CSI ≥40 group had lacrimation, aural fullness, nasal blockage, and rhinorrhea, which are cranial autonomic parasympathetic symptoms, significantly more frequently than those in the CSI < 40 group.

**Conclusions:**

Migraine patients with CASs showed significantly greater central sensitization than those without such symptoms. In particular, cranial parasympathetic symptoms were more frequent in centrally sensitized patients than in nonsensitized patients, suggesting that cranial parasympathetic activation may contribute to the maintenance of central sensitization.

**Trial registration:**

This study was retrospectively registered with UMIN-CTR on 29 Aug 2020 (UMIN000041603).

## Background

Migraine is a disabling primary headache disorder with not only head pain but also various neurological symptoms [1]. Cranial autonomic symptoms (CASs), such as lacrimation, conjunctival injection, and nasal congestion, are important clinical characteristics of trigeminal autonomic cephalalgias (TACs). They also are part of the diagnostic criteria of TACs included in the third edition of international classification of headache disorders (ICHD-3) [1]. However, CASs are not mentioned in the migraine diagnostic criteria, despite such symptoms being quite common during migraine attacks, regardless of ethnicity [2–9].

In our previous study, the patients with CASs more frequently had cutaneous allodynia than those without CASs, and we suggested that CASs were associated with central sensitization (CS) [9]; however, the validated central sensitization inventory (CSI) was not used in that investigation; therefore, the assessment of CS did not have substantial evidence.

The validated CSI is a comprehensive screening tool for evaluating the symptoms of central sensitivity syndromes (CSSs). CSSs are an overlapping, similar group of syndromes that lead to hypersensitivity, including allodynia [10, 11]. CSSs includes migraine, fibromyalgia, irritable bowel syndrome, chronic fatigue syndrome, temporomandibular joint disorder, restless leg syndrome, and multiple chemical sensitivities. Especially in migraine, nearly 80% of patients are reported to experience allodynia; migraine is therefore recognized as one of the main components of CSSs [11–14]. Other syndromes, such as chronic widespread pain, interstitial cystitis, endometriosis, vulvodynia, and chronic pelvic pain, are also included among CSSs. Furthermore, chronic musculoskeletal pain conditions, such as persistent neck pain and low back pain, are also known to be observed with hypersensitivity induced by CS [11, 14]. CSSs have many common features, such as pain, fatigue, insomnia, anxiety and depression, suggesting the possibility that they are at least partly induced by a common root etiology of CS [10].

In the present study, we investigated a new independent cohort to obtain substantial evidence concerning the relationship between CS and CASs and evaluated CS severity in migraine using the validated Japanese version of the CSI [11]. We hypothesized that the CS severity would be greater in migraine patients with CASs than in those without CASs.

## Methods

In this prospective cross-sectional study, we investigated episodic and chronic migraine patients who were seen at the headache center of Tominaga Hospital from July 2018 to December 2019. The inclusion criteria were inpatients or outpatients 20 to 79 years old who met the criteria of ICHD-3 (beta) for 1.1 Migraine without Aura, 1.2 Migraine with Aura and 1.3 Chronic Migraine [15]. Patients who also had medication overuse headache (MOH) were eligible. We excluded secondary headache patients except for MOH. TACs patients were also excluded.

On Visit 1, written informed consent for inclusion in the study was obtained from each patient. After the patients agreed, they received a questionnaire investigating their clinical features of migraine, including CASs. The questionnaire had a structured format and included CASs according to the criteria in ICHD-3 (beta): a) conjunctival injection, b) lacrimation, c) nasal congestion, d) rhinorrhea, e) eyelid edema, f) forehead and facial sweating, g) forehead and facial flushing, h) sensation of fullness in the ear, i) miosis, and j) ptosis. The participants also received the CSI form, which consists of two sections (Parts A and B). Part A assesses 25 health-related symptoms common to CSSs, with each response rated on a scale of Never (0), Rarely (1), Sometimes (2), Often (3) or Always (4), giving a potential total score of 0–100. CS severity was defined in accordance with the CSI score as follows: subclinical (0–29), mild (30–39), moderate (40–49), severe (50–59) and extreme (60–100) [16]. Part B assesses whether or not one or more specific disorders of CSSs, as well as related disorders, such as anxiety and depression, have been diagnosed previously by a physician [10, 11].

Before Visit 2, patients were requested to complete these questionnaires. They were instructed to answer the questions about CASs during the interictal period after the migraine attack. On Visit 2, one of the headache specialists (TT, DD, SK, or KI) carefully re-checked each filled-out questionnaire with the patient to reconfirm their symptoms. We stressed that it was critical for the patients to ensure that only CASs associated with headache attacks were included.

### Statistical analyses

The data were pooled in a datasheet using the Excel software program (Microsoft Excel, Microsoft Corporation, Redmond, WA, USA). We used the Kolmogorov-Smirnov test to assess the normality assumption. We checked the homoscedasticity for two and three groups using the F-test and Bartlett’s test, respectively. We evaluated the differences between two groups using an independent sample *t*-test. To compare the means of three groups and perform an ad hoc analysis, a one-way analysis of variance and Tukey’s test were used, respectively. Nominal variables associations were evaluated by the χ^2^ test. Each analysis was conducted with a threshold *P*-value of 0.05 (2-tailed) using the EZR software program [17].

The Tominaga Hospital Ethics Committee approved the protocol. We obtained written informed consent from all participants.

## Results

One hundred and sixty-five patients met the eligibility criteria; however, one patient refused to participate in this study. Therefore, 164 migraineurs were enrolled. The mean age was 41.8 (range: 20 to 77) years old. One hundred and thirty-one patients (78.9%) were women. Fifty-three patients had episodic migraine (EM), and 111 had chronic migraine (CM) (Table 1).

**Table 1:** Patient demographics (*n* = 164)

Age at study (years ± SD)		41.8 ± 13.0
		**Cases (%)**
**Females**		**131 (78.9%)**
**EM**	**MO**	**42 (25.6%)**
	**MO with MA**	**9 (5.5%)**
	**Exclusive MA**	**2 (1.2%)**
**CM with MOH**		**67 (40.9%)**
**CM without MOH**		**44 (26.8%)**

*EM* episodic migraine, *MO* migraine without aura, *MA* migraine with aura, *CM* chronic migraine, *MOH* medication overuse headache

The average score of the CSI (Part A) was 34.2 (standard deviation [SD]: 13.1). Moderate to severe sensitization was noted in 47 out of 164 patients (28.7%), and 6 patients (3.7%) had extreme central sensitization. In terms of the diagnosis of CSSs (Part B), all patients had migraine due to the inclusion criteria, and temporomandibular joint disorder and irritable bowel syndrome were noted in 20 patients (12.2%) and 17 patients (10.4%), respectively. Anxiety or panic attacks and depression were also reported in 18 patients (11.0%) and 31 patients (18.9%), respectively (Table 2).

**Table 2:** Prevalence rates of CS severity and frequency of diagnoses

	N (%)
**Central Sensitization Inventory: Part A (CSI score)**	
**Complete cases**	**164/164 (100.0)**
**Subclinical (0–29)**	**62/164 (37.8)**
**Mild (30–39)**	**49/164 (29.9)**
**Moderate (40–49)**	**30/164 (18.3)**
**Severe (50–59)**	**17/164 (10.4)**
**Extreme (> 60)**	**6/164 (3.7)**
**Central Sensitization Inventory: Part B (Diagnoses)**	
**Complete cases**	**162/164 (98.8)**
**Restless leg syndrome**	**6/164 (3.7)**
**Chronic fatigue syndrome**	**2/164 (1.2)**
**Fibromyalgia**	**2/164 (1.2)**
**Temporomandibular joint disorder**	**20/164 (12.2)**
**Migraine or tension headaches**	**164/164 (100.0)** ^a^
**Irritable bowel syndrome**	**17/164 (10.4)**
**Multiple chemical sensitivities**	**0/164 (0.0)**
**Neck injury (including whiplash)**	**13/164 (7.9)**
**Anxiety or panic attacks**	**18/164 (11.0)**
**Depression**	**31/164 (18.9)**

*CS* central sensitization, *CSI* central sensitization inventory

^a^We failed to collect the Part B questionnaire from 2 patients; however, all cases were migraine cases; hence, the number of ‘Migraine or tension headaches’ was 164

**Fig. 1:**
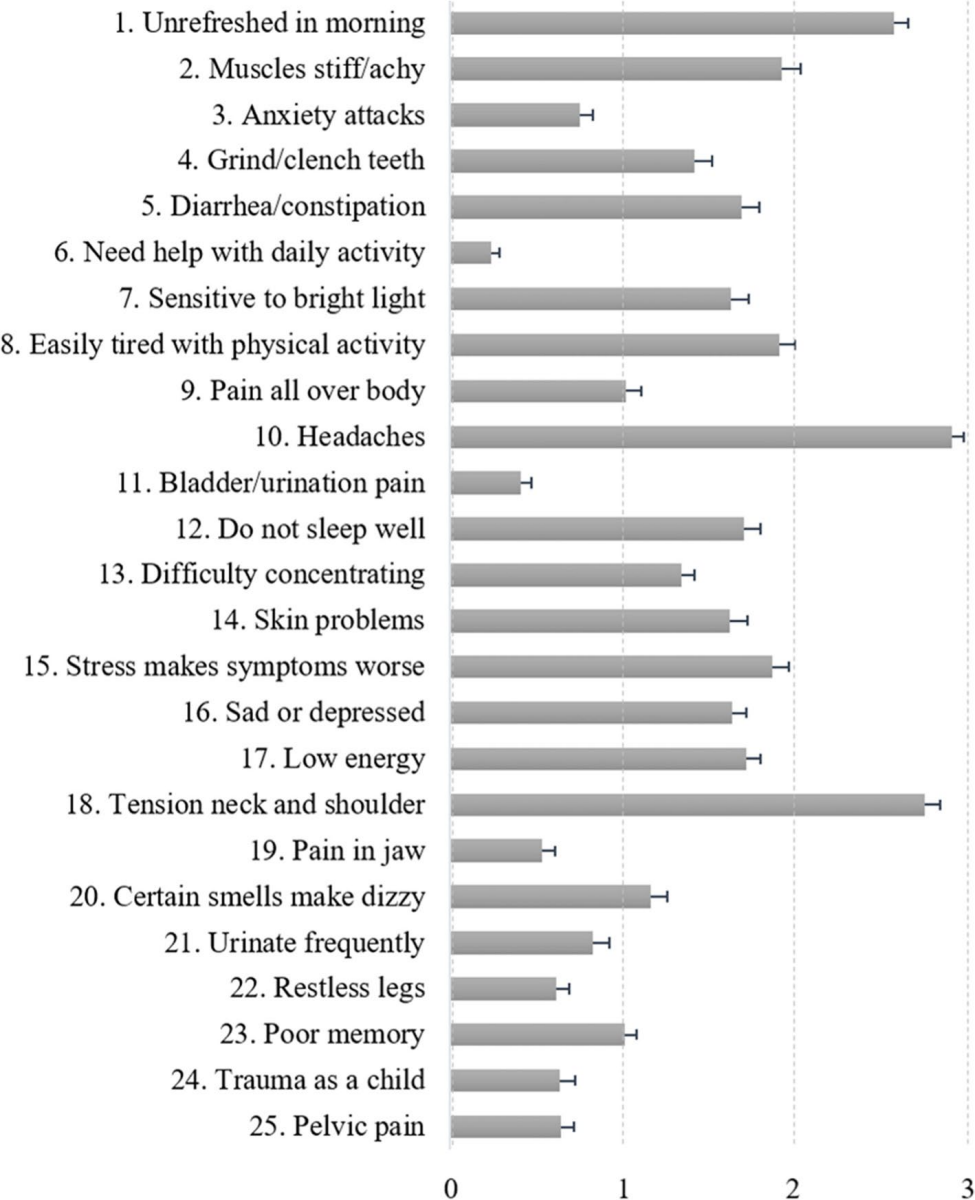
The average score for each item of the central sensitization inventory: Part A (*n* = 164). Each item is listed from 1 to 25, in accordance with the original inventory [10]. Items were measured on a 5-point temporal Likert scale with the numeric rating scale as follows: 0-Never, 1-Rarely, 2-Sometimes, 3-Often and 4-Always. The cumulative score ranges from 0 to 100. The error bars show the standard error of the items [10, 11]

The breakdown and average scores of 25 health-related symptoms are shown in Fig. 1. In addition to headache, the items ‘unrefreshed in the morning’, ‘muscle stiffness’, ‘diarrhea/constipation’, ‘sensitive to light’, ‘easily tired’, ‘do not sleep well’, ‘stress makes symptoms worse’, ‘energy is low’, ‘sad/depressed’, and ‘tension in the neck/shoulder’ showed relatively high ratings.

The CSI score of CM was higher than that of EM; however, the difference did not reach statistical significance (35.6 vs. 31.4, *p* = 0.054). We also detected no significant differences in the CSI score between, MOH and non-MOH, unilateral headache and bilateral headache, or pulsating and non-pulsating. The CS severity was relatively mild in the patients whose numerical rating scale (NRS) was < 8, even during their maximum attack (CSI 30.7). The CSI score in the patients with an attack duration exceeding 72 h during their maximum attack was significantly higher than in patients with a duration < 24 h (42.7 vs. 30.6, *p* = 0.004) (Table 3).

**Table 3:** CSI score: headache background and characteristics

		Cases (%)	CSI score ± SD	***p*** value
**Headache background**				
**Chronification**	**Complete cases**	**164/164 (100.0)**		
	**CM**	**111/164 (67.7)**	**35.6 ± 13.4**	**0.054**
	**EM**	**53/164 (32.3)**	**31.4 ± 12.3**	
**Medication overuse**	**Complete cases**	**164/164 (100.0)**		
	**MOH**	**68/164 (41.5)**	**35.3 ± 14.7**	**0.378**
	**non-MOH**	**96/164 (58.5)**	**33.5 ± 11.9**	
**Aura**	**Complete cases**	**164/164 (100.0)**		
	**with aura**	**27/164 (16.5)**	**37.1 ± 13.6**	**0.210**
	**without aura**	**137/164 (83.5)**	**33.7 ± 13.0**	
**Illness duration**	**Complete cases**	**160/164 (97.6)**		
	**< 5 years**	**11/164 (6.7)**	**35.4 ± 11.1**	**0.939**
	**5–10 years**	**18/164 (11.0)**	**33.8 ± 17.2**	
	**> 10 years**	**131/164 (79.9)**	**33.9 ± 12.8**	
**Sex**	**Complete cases**	**164/164 (100.0)**		
	**Male**	**33/164 (20.1)**	**31.1 ± 14.2**	**0.124**
	**Female**	**131/164 (79.9)**	**35.0 ± 12.8**	
**Headache characteristics**				
**Pain location**	**Complete cases**	**159/164 (97.0)**		
	**Unilateral: (strictly unilateral/unilateral side variable)**	**53/164 (32.3)**	**33.7 ± 12.3**	**0.932**
	**Bilateral: (always bilateral/bilateral but sometimes unilateral)**	**106/164 (64.6)**	**33.9 ± 13.4**	
**Pain quality**	**Complete cases**	**163/164 (99.4)**		
	**Pulsating**	**110/164 (67.1)**	**33.7 ± 13.0**	**0.604**
	**Non-pulsating**	**53/164 (32.3)**	**34.9 ± 13.4**	
**Pain severity**	**Complete cases**	**162/164 (98.8)**		
Maximum attack	**NRS 8 out 10 or more**	**116/164 (70.7)**	**35.7 ± 12.7**	**0.028**^**†**^
	**Less than 8 out of 10**	**46/164 (28.0)**	**30.7 ± 13.4**	
Usual attack	**NRS 8 out 10 or more**	**15/164 (9.1)**	**33.4 ± 14.3**	**0.792**
	**Less than 8 out of 10**	**147/164 (89.6)**	**34.3 ± 12.9**	
**Attack duration**	**Complete cases**	**152/164 (92.7)**		
**Maximum**	**< 24 h**	**61/164 (37.2)**	**30.6 ± 11.7**	**0.004**^**††**^
	**24–72 h**	**76/164 (46.3)**	**34.5 ± 13.4**	
	**> 72 h**	**15/164 (9.1)**	**42.7 ± 13.7**	
	**Post hoc: < 24 h vs. > 72 h**			**0.004**^**††**^
**Usual**	**< 24 h**	**104/164 (63.4)**	**32.2 ± 12.1**	**0.077**
	**24–72 h**	**45/164 (27.4)**	**37.0 ± 14.9**	
	**> 72 h**	**3/164 (1.8)**	**41.0 ± 13.1**	
**Usual headache frequency (moderate to severe)**	**Complete cases**	**139/164 (84.8)**		
	**< 1 day/month**	**12/164 (7.3)**	**31.1 ± 15.1**	**0.069**
	**1–10 days/month**	**106/164 (64.6)**	**33.8 ± 12.5**	
	**> 10 days/month**	**21/164 (12.8)**	**40.5 ± 15.9**	

*CSI* central sensitization inventory, *SD* standard deviation, *CM* chronic migraine, *EM* episodic migraine, *MOH* medication overuse headache, *NRS* Numerical Rating Scale

^†^*p* < 0.05 ^††^*p* < 0.01

**Fig. 2:**
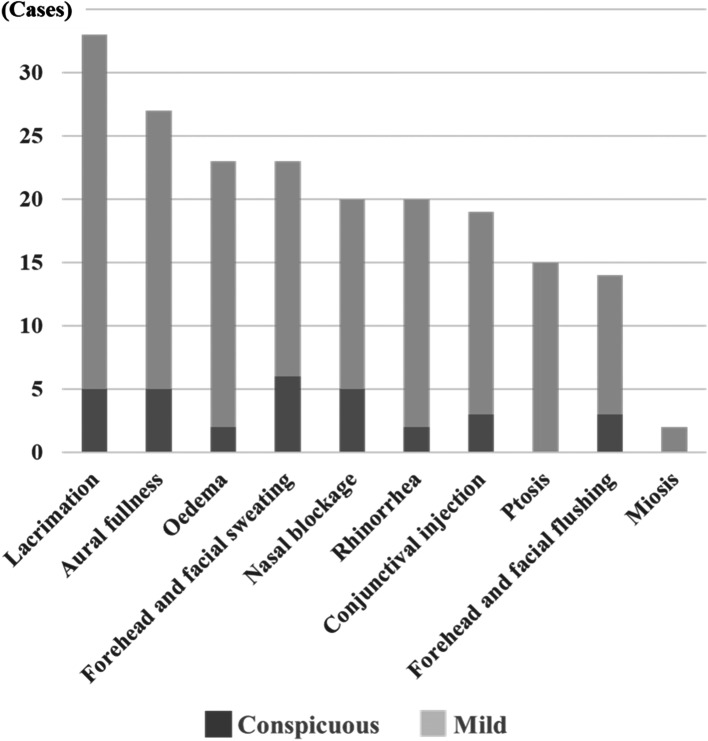
The details of cranial autonomic symptoms (*n* = 86). Dark-gray bars show conspicuous symptoms. The most frequent cranial autonomic symptom was lacrimation, followed by aural fullness, oedema, forehead and facial sweating, nasal blockage, rhinorrhea and conjunctival injection. Most of the symptoms were mild

**Fig. 3:**
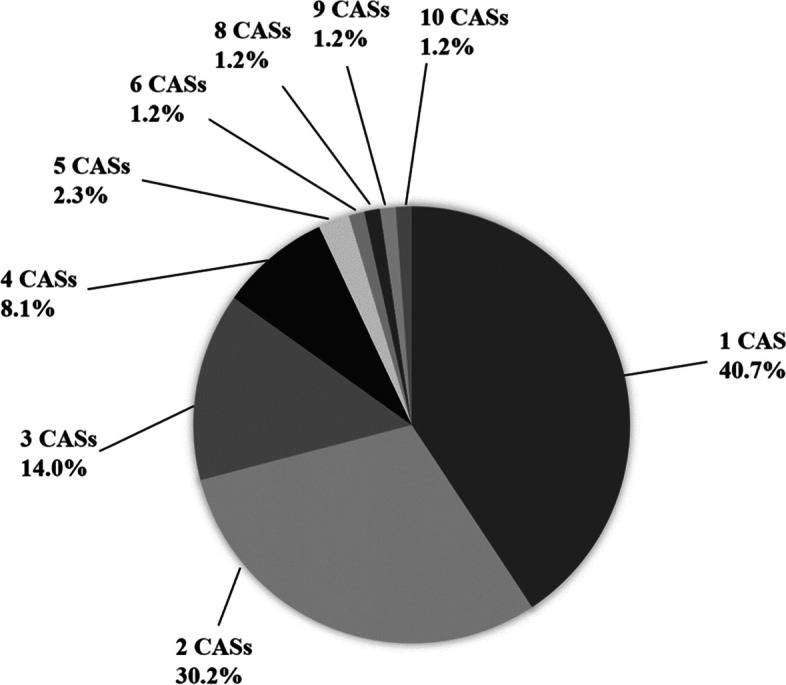
The number of cranial autonomic symptoms (*n* = 86). Seventy percent of the patients with CASs had fewer than three autonomic symptoms. CASs: cranial autonomic symptoms

Eighty-six of the 164 (52.4%) patients had at least 1 CAS. Regarding the details of the CASs, lacrimation was most common, being seen in 33 (20.1%) patients, followed by aural fullness in 27 (16.5%) patients, eyelid edema in 23 (14.0%) patients, forehead and facial sweating in 23 (14.0%) patients, nasal blockage in 20 (12.2%) patients, rhinorrhea in 20 (12.2%) patients, and conjunctival injection in 19 (11.6%) patients. Most of the cases had mild symptoms (Fig. 2). Among the patients who had CASs, 70.9% had < 3 autonomic symptoms (Fig. 3).

The CSI score of the patients with ≥3 CASs reflected a moderate severity and was significantly higher than in those without CASs (41.9 vs. 30.7, *p* = 0.0005). The score of the patients with ≥1 conspicuous CAS also reflected a moderate severity and was significantly higher than in those without such symptoms (40.7 vs. 33.2, *p* = 0.013). The patients with ‘CAS consistency >80%’ and ‘unilateral CASs’ had a higher CSI score than those without them (46.8 and 40.5 respectively); however, the difference was not significant (Table 4).

**Table 4:** CSI score: cranial autonomic symptoms

		Cases (%)	CSI score ± SD	*p* value
**Cranial autonomic symptoms**				
**Number of CAS**	**Complete cases**	**164/164 (100)**		
	**0**	**78/164 (47.6)**	**30.7 ± 10.9**	**0.000**^**††**^ **(0.00047)**
	**1–2**	**61/164 (37.2)**	**35.7 ± 13.4**	
	**3–10**	**25/164 (15.2)**	**41.9 ± 15.3**	
	**Post hoc: 0 vs. 3–10**			**0.000**^**††**^ **(0.00048)**
**At least one conspicuous CAS**	**Complete cases**	**164/164 (100)**		
	**Yes**	**22/164 (13.4)**	**40.7 ± 16.2**	**0.013**^**†**^
	**No**	**142/164 (86.6)**	**33.2 ± 12.4**	
**CAS consistency (86 cases)**	**Complete cases**	**65/86 (75.6)**		
	**< 30%**	**17/86 (19.8)**	**36.8 ± 17.1**	**0.129**
	**30–80%**	**37/86 (43.0)**	**38.1 ± 12.2**	
	**> 80%**	**11/86 (12.8)**	**46.8 ± 11.7**	
**CAS laterality** **(86 cases)**	**Complete cases**	**82/86 (95.3)**		
	**Unilateral**	**35/86 (40.7)**	**40.5 ± 13.1**	**0.056**
	**Bilateral**	**47/86 (54.7)**	**34.5 ± 14.5**	

*CSI* central sensitization inventory, *SD* standard deviation, *CAS* cranial autonomic symptoms

^†^*p* < 0.05 ^††^*p* < 0.01

The patients in the CSI ≥40 group more frequently had CASs of lacrimation, aural fullness, nasal blockage and rhinorrhea than those in the CSI < 40 group (Table 5). All of the CASs that were more frequent in the CSI ≥40 group were cranial autonomic parasympathetic symptoms (CApSs). In terms of bothersome symptoms, phonophobia, osmophobia, and allodynia were more frequent in the CSI ≥40 group than in the CSI < 40 group. The rate of interictal photophobia was also significantly higher in the CSI ≥40 group than in the CSI < 40 group (49.1 vs. 22.5, *p* = 0.001) (Table 6).

**Table 5:** Cranial autonomic symptoms

	CSI ≥ 40	CSI < 40	***p*** value
**Lacrimation**	**Complete cases**	**164/164 (100.0)**	
	**16/53 (30.2%)**	**17/111 (15.3%)**	**0.044**^**†**^
**Aural fullness**	**Complete cases**	**164/164 (100.0)**	
	**14/53 (26.4%)**	**13/111 (11.7%)**	**0.032**^**†**^
**Oedema**	**Complete cases**	**164/164 (100.0)**	
	**11/53 (20.8%)**	**12/111 (10.8%)**	**0.140**
**Forehead and facial sweating**	**Complete cases**	**164/164 (100.0)**	
	**9/53 (17.0%)**	**14/111 (12.6%)**	**0.608**
**Nasal blockage**	**Complete cases**	**164/164 (100.0)**	
	**11/53 (20.8%)**	**9/111 (8.1%)**	**0.039**^**†**^
**Rhinorrhea**	**Complete cases**	**164/164 (100.0)**	
	**12/53 (22.6%)**	**8/111 (7.2%)**	**0.010**^**†**^
**Conjunctival injection**	**Complete cases**	**164/164 (100.0)**	
	**8/53 (15.1%)**	**11/111 (9.9%)**	**0.478**
**Ptosis**	**Complete cases**	**164/164 (100.0)**	
	**6/53 (11.3%)**	**9/111 (8.1%)**	**0.706**
**Forehead and facial flushing**	**Complete cases**	**164/164 (100.0)**	
	**7/53 (13.2%)**	**7/111 (6.3%)**	**0.238**
**Miosis**	**Complete cases**	**164/164 (100.0)**	
	**2/53 (3.8%)**	**0/111 (0.0%)**	**0.194**

*CSI* central sensitization inventory

^†^*p* < 0.05 ^††^*p* < 0.01

**Table 6:** Migrainous symptoms

	CSI ≥ 40	CSI < 40	***p*** value
**Migrainous symptoms**			
**Photophobia**	**Complete cases**	**164/164 (100.0)**	
	**36/53 (67.9%)**	**70/111 (63.1%)**	**0.664**
**Interictal photophobia**	**Complete cases**	**164/164 (100.0)**	
	**26/53 (49.1%)**	**25/111 (22.5%)**	**0.001**^**††**^
**Phonophobia**	**Complete cases**	**164/164 (100.0)**	
	**41/53 (77.4%)**	**67/111 (60.4%)**	**0.049**^**†**^
**Osmophobia**	**Complete cases**	**164/164 (100.0)**	
	**32/53 (60.4%)**	**37/111 (33.3%)**	**0.002**^**††**^
**Nausea**	**Complete cases**	**164/164 (100.0)**	
	**39/53 (73.6%)**	**81/111 (73.0%)**	**1.000**
**Vomiting**	**Complete cases**	**164/164 (100.0)**	
	**22/53 (41.5%)**	**40/111 (36.0%)**	**0.614**
**Motion sensitivity**	**Complete cases**	**164/164 (100.0)**	
	**41/53 (77.4%)**	**81/111 (73.0%)**	**0.681**
**Cutaneous allodynia**	**Complete cases**	**164/164 (100.0)**	
	**18/53 (34.0%)**	**20/111 (18.0%)**	**0.039**^**†**^

*CSI* central sensitization inventory

^†^*p* < 0.05 ^††^*p* < 0.01

### Missing data

Some patients were unable to recall their illness duration, headache characteristics or CASs characteristics, so we stated the number of complete cases for each item in the tables. We also failed to collect the Part B questionnaire from 2 patients; however, all cases were migraine cases, therefore the number of ‘Migraine or tension headaches’ was 164.

## Discussion

### CSI

CS is a physiological phenomenon in which the central circuits become hypersensitive to both harmful and non-harmful stimuli [18]. Quantitative Sensory Testing (QST) is a direct CS measurement tool including static and dynamic examinations; however, its applicability in clinical practice remains low due to its high cost [10, 11].

The validated CSI consists of Part A to assess 25 health-related symptoms and Part B inquiring regarding previous diagnoses with major CSSs as well as related disorders of anxiety and depression [10].. In a previous study investigating the patients who were referred to an interdisciplinary pain clinic, 74% had CSSs, and the most frequent diagnosis was migraine/tension-type headache. A receiver operating characteristic (ROC) analysis in the study showed that the cut-off CSI score was 40 for distinguishing patients with CSSs from the controls, with a sensitivity of 81% and specificity of 75% [18]. The validated severity levels for CSI were also established in another study analyzing 167 patients with CSSs, of whom 58 were diagnosed with migraine/tension-type headache [16]. Furthermore, in a study using the CSI as a tool to evaluate CS in migraine, they found that migraine patients with CS had higher rates of RLS than those without CS, and migraine patients were three times more likely to have CS than healthy subjects [13]. These findings suggest that the CSI is a useful and practical tool for evaluating CS in CSSs, including migraine.

### CASs and CS

In this study, we found that migraine patients with ‘at least three CASs’ or ‘at least one conspicuous CAS’ had a moderate level of CS, and their centrally sensitized levels were significantly higher than in those without these issues. It was reported at a tertiary headache center in Italy that the patients with CASs more frequently had allodynia and photophobia, which are related to CS, than those without CASs [4]. In our previous study, CAS patients were also found to have allodynia more frequently than those without CASs (31.6% vs. 17.2%, *p* = 0.001); however, no significant differences were reported in the rates of throbbing pain or motion sensitivity as signs of peripheral sensitization [9]. Given these findings, CASs in migraine may induce central sensitization.

### Cranial parasympathetic contributions to CS

Among the CASs in ICHD-3 beta, forehead and facial sweating and forehead and facial flushing are caused by the sympathetic nervous system at least in some part of their mechanisms, while miosis and ptosis are considered the results of secondary sympathetic hypofunction. The CApSs, namely conjunctival injection, lacrimation, oedema, nasal blockage, rhinorrhea, and aural fullness, are the symptoms induced by parasympathetic activation. In a study establishing a numerical scale evaluating CApSs, these symptoms appeared in about 80% of the patients with CM during headache attacks, and the CApS numerical scale was concluded to be useful for evaluating parasympathetic activation [5].

In terms of pathophysiology of CASs in migraine, pain signals from the trigeminal nerve (the fifth cranial nerve: CN V) first activate the dorsal raphe nuclei (DRN) and periaqueductal gray (PAG) in the brainstem. They are then transmitted to the locus coeruleus (LC) in the ipsilateral pons, and the activated LC stimulates the ipsilateral superior salivatory nucleus (SSN). Some nerves from the CN V also stimulate the ipsilateral SSN directly, and this causes the activation of the efferent pathway of the trigeminal-autonomic reflex passing through the sphenopalatine ganglion (SPG) [19]. In a previous study, most of the severe migraine cases developed cutaneous allodynia, and SPG block using lidocaine decreased the pain but did not relieve the allodynia. These results indicated that increased cranial parasympathetic signals activated the nociceptors of the dural vessels, and these activated nociceptors induced the head pain and CS; however, the increased parasympathetic signals did not maintain CS [20].

Interestingly, in our study, the rates of miosis and ptosis, which are cranial autonomic sympathetic symptoms, did not differ markedly between the CSI ≥40 and CSI < 40 groups. In contrast, all CApSs except for conjunctival injection were significantly frequent in the patients with CSI ≥40 than in those with CSI < 40 (Table 5). These findings may be interpreted in a couple of ways: trigemino-vascular system (TVS) activation induced by recurrent pain stimulation may induce CS and activation of the cranial parasympathetic system in parallel; alternatively, cranial parasympathetic system activation induced by the activated TVS may cooperate with recurrent pain stimulation to induce CS. We further speculated that the TVS and cranial parasympathetic system may become sensitized in parallel during the course of a long migraine history, and the hyperexcitability of both systems may activate each other and maintain CS. However, further studies will be needed to elucidate the true contribution of CApSs to CS.

### CS and responsiveness to triptans

Migraine patients who never developed allodynia were reported to respond well to triptans [21]. It is also reported that chronic migraine patients had lower pain thresholds than episodic migraine patients, and CS is considered to be one of the reasons for non-responsiveness to triptans [22]. On the other hand, migraine patients with CASs have reported to respond to triptans well, and the presence of CASs can be a predictor of the responsiveness to triptans [23, 24]. Furthermore, in a study of migraine patients, 5 out of 10 responders to rizatriptan had at least 1 CAS, which were not reported in the 10 non-responders. Responders also showed higher levels of calcitonin gene-related peptide (CGRP) which is a potential trigeminal marker, and in the patients with CASs, the parasympathetic marker of vasoactive intestinal polypeptide (VIP) was detected at baseline, the levels of which were reduced after rizatriptan administration [25]. The authors speculated that the trigemino-parasympathetic reflex was over-activated in migraine patients with CASs, and the activation strongly recruited the peripheral neurovascular 5-HT1B/1D receptors that are the target of triptans. We therefore identified an apparent paradox between the fact that CS in migraine was found to be related to non-responsiveness to triptans and that migraine with CASs was found to be related to CS. In the present study, we did not investigate the responsiveness to triptans; however, one possible interpretation is that there might be some type of CS (e.g. allodynia-dominant type, CAS-dominant type, etc.) that depends on the extent of sensitization. Further prospective studies will be needed in order to clarify the triptan responses in migraine patients with CASs in accordance with the CSI level and thereby our understanding of the underlying mechanism of CS.

### Strengths and limitations

The strength of the present study is that we obtained substantial evidence supporting the relationship between CS and CASs using the validated CSI in a relatively large sample of migraineurs for the first time.

However, several limitations associated with the present study warrant mention. All of the data were collected in the interictal periods; therefore, there might have been some recall bias. Another limitation is the potential reporting bias; for instance, ptosis or forehead and facial flushing may have been difficult for patients to recognize. Furthermore, this study was conducted at a tertiary headache center, therefore there may have been some referral bias, which might have led to the inclusion of a cohort consisting of severe migraineurs. Aural fullness was included in the criteria of ICHD-3 (beta) and removed in the latest ICHD-3 criteria; however, aural fullness was reported to be a very common CAS during migraine attacks in previous studies [5, 8, 9]. We therefore used the CAS criteria of ICHD-3 (beta), and consequently, aural fullness was found to be the second-most common symptom in the present study. Further studies will be needed in order to clarify the pathophysiological implications of aural fullness during migraine attacks. Finally, the CSI includes an item evaluating osmophobia, which might have led to the high rate of osmophobia in the CSI ≥40 group; however, the findings of the present and previous studies support the hypothesis that osmophobia is associated with CS [9, 26, 27].

## Conclusions

This was the first study to investigate CS in migraine with CASs using a validated CSI, with findings suggesting that the presence of CASs in migraine is related to CS. We also found that CApSs in particular were more frequently observed in migraineurs with CS than in those without CS and speculated that TVS and the cranial parasympathetic system were sensitized in parallel, potentially contributing to the maintenance of CS. The treatment of migraine patients with heavy burdens is still insufficient, and CS is recognized as one reason for non-responsiveness to the treatments [22, 28, 29]. Prospective studies are therefore needed to clarify which specific treatments can improve the CS severity and CAS occurrence rate so that clinicians can provide more appropriate treatment for migraine patients.

## Data Availability

The datasets used in the present study are available from the corresponding author, upon reasonable request.

## Abbreviations

CApSs: Cranial autonomic parasympathetic symptoms
CASs: Cranial autonomic symptoms
CGRP: Calcitonin gene-related peptide
CM: Chronic migraine
CN V: Fifth cranial nerve
CS: Central sensitization
CSI: Central sensitization inventory
CSS: Central sensitivity syndrome
DRN: Dorsal raphe nuclei
EM: Episodic migraine
ICHD-3: The international classification of headache disorders 3rd edition
LC: Locus coeruleus
MA: Migraine with aura
MO: Migraine without aura
MOH: Medication overuse headache
NRS: NUMERICAL rating scale
PAG: Periaqueductal gray
QST: Quantitative sensory testing
ROC: Receiver operating characteristic
SD: Standard deviation
SPG: Sphenopalatine ganglion
SSN: Superior salivatory nucleus
TACs: Trigeminal autonomic cephalalgias
TVS: Trigemino-vascular system
VIP: Vasoactive intestinal polypeptide
